# Significance of Free Convection Flow over an Oscillating Inclined Plate Induced by Nanofluid with Porous Medium: The Case of the Prabhakar Fractional Approach

**DOI:** 10.3390/mi13112019

**Published:** 2022-11-19

**Authors:** Ali Raza, Umair Khan, Sayed M. Eldin, Abeer M. Alotaibi, Samia Elattar, Ballajja C. Prasannakumara, Nevzat Akkurt, Ahmed M. Abed

**Affiliations:** 1 Department of Mathematics, University of Engineering and Technology, Lahore 54890, Pakistan; 2 Department of Mathematics, Minhaj University, Lahore 54770, Pakistan; 3 Department of Mathematical Sciences, Faculty of Science and Technology, Universiti Kebangsaan Malaysia, UKM, Bangi 43600, Selangor, Malaysia; 4 Department of Mathematics and Social Sciences, Sukkur IBA University, Sukkur 65200, Pakistan; 5 Center of Research, Faculty of Engineering, Future University in Egypt, New Cairo 11835, Egypt; 6 Department of Mathematics, Faculty of Science, University of Tabuk, P.O. Box 741, Tabuk 71491, Saudi Arabia; 7 Department of Industrial & Systems Engineering, College of Engineering, Princess Nourah Bint Abdulrahman University, P.O. Box 84428, Riyadh 11671, Saudi Arabia; 8 Department of Studies and Research in Mathematics, Davangere University, Davangere 577002, Karnataka, India; 9 Department of Rare Earth Elements Application and Research Center, Munzur University, 62000 Tunceli, Turkey; 10 Department of Industrial Engineering, College of Engineering, Prince Sattam Bin Abdulaziz University, Al-kharj 16273, Saudi Arabia; 11 Industrial Engineering Department, Faculty of Engineering, Zagazig University, Zagazig 44519, Egypt

**Keywords:** nanofluid, heat transfer, Brinkman-type fluid, Prabhakar fractional derivative, slip effects, Newtonian heating effect

## Abstract

Given the importance and use of electrically conducted nanofluids, this work aims to examine an engine-oil-based nanofluid including various nanoparticles. In the current study, a fractional model for inspecting the thermal aspect of a Brinkman-type nanofluid, composed of (molybdenum disulfide (*MOS*_2_) and graphene oxide (*GO*) nanoparticles flows on an oscillating infinite inclined plate, which characterizes an asymmetrical fluid flow, heat, and mass transfer. Furthermore, the Newtonian heating effect, magnetic field, and slip boundary conditions were taken into account. The objectives for implementing the Prabhakar-like fractional model are justified because this fractional algorithm has contemporary definitions with no singularity restrictions. Furthermore, the guided fractional model was solved using the Laplace transform and several inverse methods. The obtained symmetrical solutions have been visually analyzed to investigate the physics of several relevant flow parameters on the governed equations. Some exceptional cases for the momentum field are compiled to see the physical analysis of the flowing fluid symmetry. The results show that the thermal enhancement can be progressively improved with the interaction of the molybdenum disulfide-engine oil-based nanofluid suspension, rather than with the graphene oxide mixed nanoparticle fluid. Furthermore, the temperature and momentum profiles enhance due to the factional parameters for molybdenum disulfide and the graphene oxide-engine oil-based nanofluid suspension. This study’s graphical and numerical comparison with the existing literature has shown a very close resemblance with the present work, which provides confidence that the unavailable results are accurate. The results show that an increase improved the heat transmission in the solid nanoparticle volume fractions. In addition, the increment in the mass and heat transfer was analyzed in the numerical evaluation, while the shear stress was enhanced with the enhancement in the Prabhakar fractional parameter *α*.

## 1. Introduction

Due to the numerous uses for thermal energy across all industries, such as the bio-medical and engineering fields, and the current exceptionally high demand for thermal energy, researchers have been motivated to accelerate the rate of thermal perturbation from the pending sources by the extraordinary vision of nano-science. Enhancement in heat effectiveness can have an advantage over solicitations incorporating the analysis of plasma, microelectronic equipment, such as nuclear reactors, space refrigeration, microchips in computers, power generation, and many more [1]. Non-Newtonian fluids possess broad applications in science and technology, such as in cosmetics, biochemical, and medical applications. Because of their extensive applications in different fields, investigators are constantly fascinated by these fluids [2,3]. Therefore, different models in the past for non-Newtonian fluids are studied. One of them is known as the Brinkman-type fluid, which Darcy introduced for those fluids that pass through small permeable surfaces [4]. More precisely, this fluid model is appropriate for the flow on a body with a negligible permeability. However, Darcy’s law does not apply to the flow on a body or surfaces with a very high porosity. Using a fluid model across the porous surfaces was an idea put forth by Brinkman [5,6]. A viscous, incompressible fluid with a significant amount of porosity, is known as a Brinkman-type fluid. Minimal research in this regard, has been achieved, through the Brinkman-type fluid. In particular, convection flows with Brinkman-type fluid and heat and mass transmission are much less studied, while these investigations have abundant applications in many industries [7,8].

The sharp rise in energy claims worldwide has directed increased efforts to accomplish energy-effective arrangements. The use of innovative schemes to expand the thermal concert of heat exchangers is one of the furthermost serious and modern subjects in the modern world. However, most of these approaches are accompanied by a rise in the pressure drop, called defect. Specifically, the enhancement procedure is hydraulic and thermally supportive [9]. Recently, nano-structure carbon mixtures, such as graphite, graphene, and some metals, such as molybdenum and magnesium, have been utilized to stock hydrogen gas. Nanofluids are simply latent heat-transmitting flowing liquids with enhanced thermophysical properties, and a heat transfer execution that may be used in various devices for more efficient operations. They are used to improve the thermal conductivity of base fluids, such as oil, ethylene glycol, water, propylene glycol, and so on, because they are poor heat-transmitting fluids. Temperature reduction, cancer treatment, and manufacturing use various biomedical and scientific engineering fields. Thermic conduction of a schematic heat-transporting fluid is enriched by the interruptions of significant molecules, which increases the heat transfer coefficient. Because solid metallic components have more thermic conduction than base fluids, suspended constituents are used to promote thermic conduction and heat transmission. The significant advantages of nanofluids are that they have a sufficient viscosity and are auxiliary stable with enhanced wetting, flowing, and diffusion characteristics along the solid surfaces, even for tiny immersion nanoparticles [10]. Srivastava et al. [11] investigated a biological population fractional model. The blended homotopy methods relating to the Sumudu transform are operated to find the solutions and show that the prey’s population abruptly declines with time. Kumar et al. [12] derived a new numerical technique to obtain the numerical solution of fractional PDEs, including the Caputo–Fabrizio derivatives, compared their obtained numerical results with the analytical results, and determined that their anticipated numerical technique attains accurate results. Mahanthesh [13] numerically studied the nanofluid flow and heat transfer using the modified Buongiorno model. The author proved that the interruption of the nanoparticle’s growth increases the thermal conduction and consequently expands the temperature. Rana et al. [14] investigated the flow of ethylene glycol-based titania nano liquid using the finite difference method, and noted that the accumulation of nanoparticles increases the temperature, and the velocity field is reduced. The finite difference scheme was used to examine the radiative flow of a polar nonliquid, along with a stretchable sheet in [15]. It was observed that the heat generation parameter has a decreasing impact on the Nusselt number. The impact of the nanoparticle combination upon the 3D flow of the titanium nonliquid, because of an exponentially extended surface, was studied in [16], by the shooting method and showed that the impact of the magnetism is much more in the ordinary fluid than in the nanofluid. Abro et al. [17] studied the influences of the magnetized nanoparticles, analytically, on the natural convection flow of the nanofluid using the Laplace method. They proved that the improvement in volume fraction increases the thickness of the thermal boundary layer. For this article’s sake, we incorporate new studies, based on the exact and numerical fractional approaches [18,19,20,21,22,23,24,25]. Shafee et al. [26] used computational methods to forecast the turbulent nanofluid (CuO/H_2_O) flow and heat transfer, and showed that the inclusion of helical tabulator measurement enhances the core nanofluid movement. Farshad and Sheikholeslami [27] discussed the turbulent nanomaterial flow using the finite volume method and showed that by enhancing friction because of the increasing inlet velocity, the quantity of the Bejan number decreases. Hussanan et al. discussed and evaluated the MHD’s inherently unstable flow of nanofluids passing through an accelerating upright plate, set in a poriferous medium [28]. Sheikholeslami et al. [29] investigated the effect of the persuaded magnetic flux on heat conduction using the KKL (Koo Kleinstreuer-Li) relationship on the nanofluid flow. Akyürek et al. [30] examined the experimentally turbulent forced convective thermal transmission, as well as the pressure drop properties of the nanofluids through a concentric tube. They proved that nanofluids with small particle concentrations did not demonstrate a significant impact on the pressure drop variation. 

Fractional calculus is an appropriate tool to explain various anomalous dynamics and processes in complex media, in physics, rheology, and electrical engineering [31,32,33,34,35,36]. Fractional calculus techniques are also applied to evaluate the anomalous relaxation phenomena [37]. In current years, it has been determined that some fractional operators and non-singular kernels are anticipated. For instance, the Atangana–Baleanu derivative and the Mittag–Leffler kernel [38] have attracted a lot of attention. A fractional derivative with a Prabhakar-like kernel is recommended by Giusti and Colombaro [39], which is the most extensive form of the Atangana–Baleanu derivative. Mahanthesh et al. [40] studied an unsteady nonlinear Casson fluid and an oscillating plate, analytically, with the Laplace technique. They proved that the nonlinear convective flow has a substantial impact on heat, as well as mass transfer properties. Abro et al. [41] studied a fractional model of the unsteady natural convection radiating flow, along with the Fourier sine transform, and showed that enhancing the fractional parameter velocity has no symmetrical behavior. Saqib et al. [42] analyzed the Brinkman-type nanofluid with CNTs and ramped the temperature conditions. Khan et al. [43] discussed an advection–reaction diffusion fractional model numerically using the Laplace, forward Euler, and Lagrange interpolation methods. They delivered a base for their considered model’s uniqueness, existence, and the HU-stability analysis. Abro et al. [44] investigated the thermal influences of the MHD micropolar fluid which was secreted by the Caputo–Fabrizio derivative. They found the exact solutions by applying the Fourier sine and the Laplace approach. They concluded that the velocity decreases while growing the magnetic parameters. Siddiqui et al. [45] studied the water-based ferrofluids, numerically, by employing a unique finite difference technique. They noted that the magnitude of the secondary vortices declines if the magnetic strength is boosted. Pandey et al. [46] discussed a space-time advection–reaction–diffusion fractional equation with the homotopy perturbation method. They presented that solute concentration when the considered system tended to move from the fractional-order to integer-order, in the existence of the sink and source terms. They claimed that their proposed method is more suitable for dealing with a fractional model, taking Mittag–Leffler’s fractional derivative and estimating the motion of the diverse models ascending in various science and engineering disciplines. Ahokposi et al. [47] recently investigated the groundwater fractal flow with the fractional differentiation and the Mittag–Leffler rule. They employed three distinct numerical strategies: implicit, explicit, and the Crank–Nicholson approach. Khan et al. [48] focused on the flow of generalized Casson fluids with the fractional derivatives, and discovered the closed-form solutions in the Wright function. Khan et al. [49] used the Caputo–Fabrizio derivatives in the heat transfer study of a Maxwell fluid in, another paper. This is an appealing research subject because of the major uses of nanofluids in cooling loads, solar thermal energy, electronic cooling, and solar energy engineering.

According to a review of the literature, as mentioned above, no studies have been carried out on the MHD Brinkman-type nanofluids (such as graphene oxide and molybdenum disulfide nanoparticles with a viscoelastic base fluid), using the Newtonian heating effect, slip boundary conditions, and our suggested setting, in combination with the Prabhakar time-fractional approach to obtain the better energy transfer phenomenon. To address this research gap, a Prabhakar time-fractional Brinkman-type fluid model for the flow of $\left(MoS_{2}\right)\;$and (GO) based nanoparticles under the influence of an externally inclined magnetic field, was investigated. According to the available literature, the integer order derivatives are local in nature, but the time-fractional derivatives are non-local and preserve the memory property. With such motivations, the current research attempts to offer Prabhakar with a fractional model for a nanofluid problem, involving multiple types of nanoparticles. A close thermal inspection of the modified nanofluid model is offered by applying molybdenum disulfide and graphene oxide nanoparticles with a viscoelastic base fluid. The comparative thermal data for these various nanoparticles is provided to further understand the energy thermal transfer mechanisms. The primary phrases and concepts of the Prabhakar fractional model are presented first, followed by the application of this novel technique in the simulated situation. In two respects, this study is innovative. It first demonstrates the application of the Prabhakar fractional model to various coupled differential systems. Second, this model confirms the thermal applications of different nanoparticles to improve the heating transportation. Furthermore, temperature, concentration, and velocity differences are depicted graphically to demonstrate the effect of the controlling factors.

## 2. Mathematical Formulation

**Figure 1:**
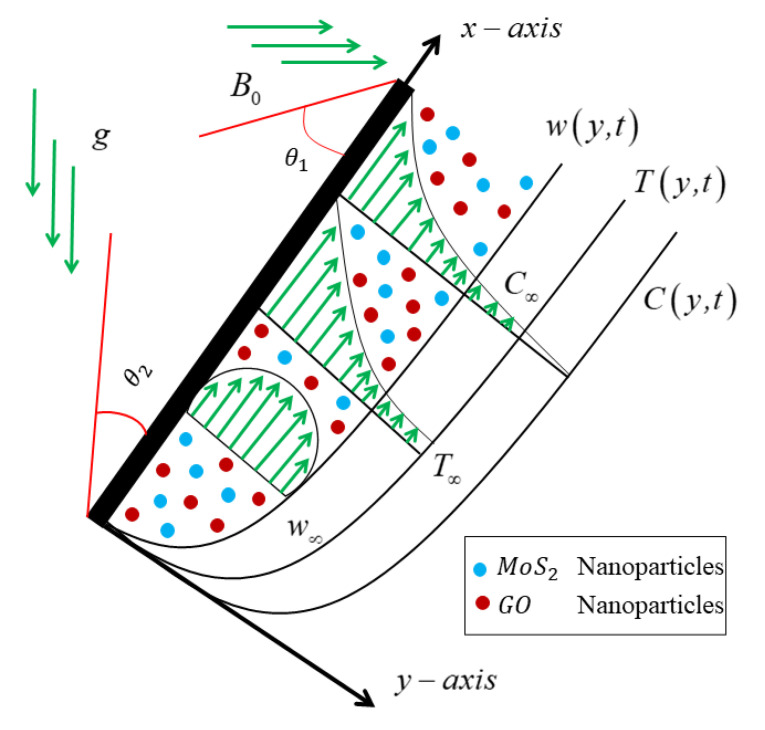
Physical model of the flow problem.

Consider a free convection Brinkman-type nanofluid, mixed with (molybdenum disulfide, graphene oxide) nanoparticles and base engine oil fluid, moving over a poured infinite plate has been shown in Figure 1. The *x*-axis is drawn vertically up the plate, whereas the *y*-axis is orthogonal to it. The pressure gradient is supposed to be ignored in the presented model, and the moving fluid is electrically conducted. An angled magnetic field, sliding, and the Newtonian heating effects are also considered. Because the magnetic Reynolds number is assumed to be very small, the induced magnetic field is ignored, the base is assumed to be fluid, and the suspension nanoparticles are in the thermal equilibrium. At t=0, both the plate and the combined fluid are at rest, with the ambient temperatures and concentrations $\;T_{\infty }$ and$\;\;C_{\infty }$. Due to the increase in the temperature variation and the oscillations of the pored oscillating plate, the free convection develops after a brief interval of time$\;t>0^{+}$, and the constant nanofluid begins to move on the inclined plate. The constant plate vibrates with the constant velocity f(t), having some of its Laplace. We made the following assumptions.

- Except for the impact of the body action term, all fundamental fluid parameters are supposed to be fix.
- An applied magnetic field with a strength of$\;B_{o}^{2}\;$is inclined with$\;\theta_{1}\;$as the inclination of the magnetic field.
- Because the fluid’s conductivity is considered low, the magnetic Reynolds number is less than one, and the induced field is small, compared to the transverse magnetic field.
- It is also assumed that the temperature, concentration, and velocity depend on y and t.
- It is also assumed that there is still no applied voltage, since the electric field is nonexistent [50].
- Based on the above presumptions and Boussinesq’s approximations [51], the governed equations can be stated as follows [42,50].

Momentum equation:

(1) $$\rho_{nf}\left(\frac{\partial w_{\left(y,t\right)}}{\partial t}+\beta_{1}^{*}w_{\left(y,t\right)}\right)\;=\mu_{nf}\frac{\partial^{2}w_{\left(y,t\right)}}{\partial y^{2}}-\sigma_{nf}B_{o}^{2}sin\left(\theta_{1}\right)w_{\left(y,t\right)}-\frac{\mu_{nf}}{k}w_{\left(y,t\right)}\;+g{\left(\rho \beta_{T}\right)}_{nf}Cos\left(\theta_{2}\right)\left(T_{\left(y,t\right)}-T_{\infty }\right)\;+g{\left(\rho \beta_{c}\right)}_{nf}Cos\left(\theta_{2}\right)\left(C_{\left(y,t\right)}-C_{\infty }\right)$$

Thermal equation:

(2) $${\left(\rho C_{p}\right)}_{nf}\frac{\partial T_{\left(y,t\right)}}{\partial t}=-\frac{\partial \delta_{\left(y,t\right)}}{\partial y}$$

Fourier law of thermal flux:

(3) $$\delta_{\left(y,t\right)}=-k_{nf}\frac{\partial T_{\left(y,t\right)}}{\partial y}$$

Diffusion balance equation:

(4) $$\frac{\partial C_{\left(y,t\right)}}{\partial t}=-\frac{\partial J_{\left(y,t\right)}}{\partial y}$$

Fick’s law:

(5) $$J_{\left(y,t\right)}=-D\frac{\partial C_{\left(y,t\right)}}{\partial y}$$

Furthermore, the identical physical conditions are specified as [52,53]:

$$w_{\left(y,0\right)}=0,\;\;T_{\left(y,0\right)}=T_{\infty },\;\;\;\;\;C_{\left(y,0\right)}=C_{\infty }\;;\;\;\;\;\;\;\;\;\;\forall \;y\geq 0$$

$$w_{\left(0,t\right)}-{h\frac{\partial w_{\left(y,t\right)}}{\partial y}|}_{y=0}=U_{o}H\left(t\right)f\left(t\right),\;\;\;\;\;{\frac{\partial T_{\left(y,t\right)}}{\partial y}|}_{y=0}=-\frac{b}{k}T_{\left(0,t\right)}\;\;\;\;\;\;\;C_{\left(0,t\right)}=C_{w};\;\;t>0$$

$$w_{\left(y,t\right)}\to 0,\;\;T_{\left(y,t\right)}\to T_{\infty },\;\;\;\;C_{\left(y,t\right)}\to C_{\infty };\;\;\;y\to \infty \;\;,\;\;\;t>0$$

where$\;w_{\left(y,0\right)}\;$is the fluid velocity, $\rho_{nf}\;$symbolizes the effective density of the nanofluid, $\mu_{nf}\;$is the effective dynamic viscosity of the nanofluid, B indicates the magnetic field, $\beta_{Tnf}$ is the effective thermal volumetric coefficient, $\beta_{Cnf}$ is the effective solutal volumetric coefficient, $k_{nf}\;$is the effective thermal conductivity, and D indicates the diffusion coefficient. The mathematical relations for$\;\rho_{f},\;\mu_{f},\;\sigma_{f},\;k_{f}\;$regular nanofluid parameters with different thermal characteristics are presented in Table 1.

The subscripts nf, f, s in the above table denote nanofluid, base fluid, and solid, all regarded as separate nanoparticles. We implement the following non-dimensional quantities to non-dimensionalize the given unsteady problem:

$$w^{*}=\frac{w}{U_{o}},\;\;\;\;y^{*}=\frac{U_{o}y}{\upsilon_{f}},\;\;\;\;t^{*}=\frac{t}{t_{o}},\;\;\;t_{o}=\frac{\nu_{f}}{U_{o}^{2}},\;\;\;\;T^{*}=\frac{T_{\left(y,t\right)}-T_{\infty }}{T_{w}-T_{\infty }},\;\;\;\;q^{*}=\frac{q}{q_{o}},\;\;J^{*}=\;\frac{J}{J_{o}},\;\;\;\;\;C^{*}=\frac{C_{\left(y,t\right)}-C_{\infty }}{C_{w}-C_{\infty }},\;\;h^{*}=\frac{U_{o}}{\nu_{f}}h,\;\;\omega^{*}=\frac{\nu_{f}\omega }{U_{o}^{2}}$$

The non-dimensional restrictions indicated above, to reduce the number of variables in the controlled Equations (1)–(5) and ignoring the “*” notation, one obtains

(6) $$\Lambda_{o}\left(\frac{\partial w_{\left(y,t\right)}}{\partial t}+\beta_{1}w_{\left(y,t\right)}\right)=\Lambda_{1}\frac{\partial^{2}w_{\left(y,t\right)}}{\partial y^{2}}-\Lambda_{2}M\;sin\left(\theta_{1}\right)w_{\left(y,t\right)}-\frac{\Lambda_{1}}{K}w_{\left(y,t\right)}+\Lambda_{3}Cos\left(\theta_{2}\right)T_{\left(y,t\right)}+\Lambda_{4}Cos\left(\theta_{2}\right)C_{\left(y,t\right)}$$

(7) $$\Lambda_{5}Pr\frac{\partial T_{\left(y,t\right)}}{\partial t}=\Lambda_{6}\frac{\partial^{2}T_{\left(y,t\right)}}{\partial y^{2}},$$

(8) $$Sc\frac{\partial C_{\left(y,t\right)}}{\partial t}=\frac{\partial^{2}C_{\left(y,t\right)}}{\partial y^{2}},$$

where the equivalent conditions are incorporated as

(9) $$w_{\left(y,0\right)}=0,\;\;T_{\left(y,0\right)}=0,\;\;\;\;\;C_{\left(y,0\right)}=0;\;\;\;\;\;\;\;\;\;\forall \;y\geq 0$$

(10) $$w_{\left(0,t\right)}-h{\frac{\partial w_{\left(y,t\right)}}{\partial y}|}_{y=0}=H\left(t\right)f\left(t\right),\;\;\;\;\;{\frac{\partial T_{\left(y,t\right)}}{\partial y}|}_{y=0}=-\left(1+T_{\left(0,t\right)}\right)\;\;\;\;\;\;\;C_{\left(0,t\right)}=1;\;\;t>0$$

(11) $$w_{\left(y,t\right)}\to 0,\;\;T_{\left(y,t\right)}\to 0,\;\;\;\;C_{\left(y,t\right)}\to 0;\;\;\;y\to \infty \;\;,\;\;\;t>0$$

Equation (9) represents the constant fluid motion, thermal profile, and concentration field in the above-transformed conditions at t=0, as supposed in the problem formulation section. In Equation (10), the rate of the fluid flow with the constant fluid velocity f(t) with slipping, the boundary effect is implemented. Furthermore, the thermal field rate with a constant concentration in the Newtonian heating effect is considered. Moreover, in the last boundary equation (11), the zero-fluid movement, temperature, and concentration at y→∞ are considered. Finally, the thermal features of the considered base fluid (engine oil) and the nanoparticles (molybdenum disulfide, graphene oxide) are examined in Table 2.

where:

$$Pr=\frac{\mu C_{p}}{\kappa_{n}},\;\;\;\;\;\beta_{1}=\frac{\upsilon_{f}\beta_{o}^{*}}{U_{o}^{2}},\;\;\;\;\;\;Gr=\frac{g{\left(\upsilon \beta_{T}\right)}_{f}\left(T_{w}-T_{\infty }\right)}{U_{o}^{3}},\;\;\;\;\;\;\;M=\frac{\sigma_{f}\upsilon_{f}B_{o}^{2}}{\rho_{f}U_{o}^{2}}\;,\;\;Sc=\frac{\upsilon_{f}}{D},\;\;\;\;K=\frac{kU_{0}}{\nu_{f}}\;\;\;\;Gm=\frac{g{\left(\upsilon \beta_{C}\right)}_{f}\left(C_{w}-C_{\infty }\right)}{U_{o}^{3}},\;\;\;\;\Lambda_{o}=\left(1-\varphi \right)+\varphi \frac{\rho_{s}}{\rho_{s}},\;\;\;\;\;\Lambda_{1}=\frac{1}{{\left(1-\varphi \right)}^{2.5}},\;\;\;\;\;\Lambda_{2}=\frac{\sigma_{nf}}{\sigma_{f}}\;\Lambda_{3}=\left(1-\varphi \right)+\varphi \frac{{\left(\rho \beta_{t}\right)}_{s}}{{\left(\rho \beta_{t}\right)}_{f}},\;\;\;\Lambda_{4}=\left(1-\varphi \right)+\varphi \frac{{\left(\rho \beta_{c}\right)}_{s}}{{\left(\rho \beta_{c}\right)}_{f}},\;\;\Lambda_{5}=\left(1-\varphi \right)+\varphi \frac{{\left(\rho C_{p}\right)}_{s}}{{\left(\rho C_{p}\right)}_{f}},\;\;\;\;\;\;\;\;\;\;\Lambda_{6}=\left(\frac{k_{s}+\left(n-1\right)k_{f}-\left(n-1\right)\left(k_{f}-k_{s}\right)\varphi }{k_{s}+\left(n-1\right)k_{f}+\left(k_{f}-k_{s}\right)\varphi }\right)$$

Signify the Prandtl number, Brinkman fluid parameter, heat Grashof number, mass Grashof number, inclined magnetic field, Schmidt number, dimensionless porosity parameter and$\;\Lambda_{o},\;\Lambda_{1},\;\Lambda_{2},\;\Lambda_{3},\;\Lambda_{4},\;\Lambda_{5},\;\Lambda_{6}\;$are the constants raised during the mathematical calculations, respectively.

## 3. Prabhakar Fractional Derivative Scheme

A practical and contemporary mathematical fractional approach has been used in this work, from which the thermal memory effect may be examined. As a result, the Prabhakar fractional derivative is presented here, which is based chiefly on the modified Fourier’s and Fick’s laws of thermal conductivity [56]

(12) δ(y,t)=−D Cα,β,αγ∂T(y,t)∂y

(13) J(y,t)=−D Cα,β,αγ∂C(y,t)∂y

where D Cα,β,αγ is the Prabhakar fractional derivative, which may be derived numerically [56,57]

D Cα,β,αγh(t)=Eα,n,−β,α−γhn(t)=∫0t(t−τ)n−β−1Eα,n−β−γ(Y(t−τ)α)hn(τ) dτ

where $h^{\left(n\right)}$ represents the nth derivative of $h\left(t\right)\in AC^{n}\left(0,b\right),\;AC^{n}\left(0,b\right)$ and means a real-valued function that has continuous derivatives of the (n−1) order with interval (0,b).

$$E_{\alpha ,\beta ,Y}^{\gamma }h\left(t\right)=\overset{t}{\underset{0}{\int }}{\left(t-\tau \right)}^{\beta -1}E_{\alpha ,\beta }^{-\gamma }\left(Y{\left(t-\tau \right)}^{\alpha }\right)h\left(\tau \right)\;d\tau$$

is known as the Prabhakar integral with

$$E_{\alpha ,\beta }^{\gamma }\left(z\right)=\overset{\infty }{\underset{m=0}{\sum }}\frac{\Gamma \left(\gamma +m\right)z^{m}}{m!\;\Gamma \left(\gamma \right)\Gamma \left(\alpha m+\beta \right)},\;\alpha ,\beta ,\gamma ,z\in \mathbb{C},\;\;\;\;\;\;Re\left(\alpha \right)>0$$

is three-dimensional, The MittagLeffler function, and $e_{\alpha ,\beta }^{\gamma }\left(\alpha ;t\right)=t^{\beta -1}E_{\alpha ,\beta }^{\gamma }\left(\alpha t^{\alpha }\right)$ is the Prabhakar kernel. The Prabhakar fractional derivative operator D Cα,β,αγ, the Laplace transform is given as

(14) ℒ[D Cα,β,Yγh(t)]=Yβ−m(1−Yζ−α)γℒ{hm(t)}

and by taking β=γ=0 the classical Fourier law can also be obtained. 

## 4. Solution of the Problem

### 4.1. Solution of the Temperature Profile

Because the criterion for is assumed to be β∈[0,1), use m=0 in the preceding formula of Equation (14). Furthermore, for the temperature field solution, the Laplace transformation scheme on Equations (7) and (12) and its accompanying conditions are used

(15) $$\Lambda_{5}Pr\;s\;{\bar{T}}_{\left(y,s\right)}=-\Lambda_{6}\frac{\partial {\bar{\delta }}_{\left(y,s\right)}}{\partial y}$$

(16) $${\bar{\delta }}_{\left(y,s\right)}=-s^{\beta }{\left(1-\alpha s^{-\alpha }\right)}^{\gamma }\frac{\partial {\bar{T}}_{\left(y,s\right)}}{\partial y}$$

(17) $${\frac{\partial {\bar{T}}_{\left(y,s\right)}}{\partial y}|}_{y=0}=-\left(\frac{1}{s}+{\bar{T}}_{\left(0,s\right)}\right);\;\;{\bar{T}}_{\left(y,s\right)}\to 0,\;y\to \infty$$

By plugging Equation (16) into (15) and using the above-transformed conditions, the thermal profile may be solved as follows

(18) $${\bar{T}}_{\left(y,s\right)}=\frac{1}{\sqrt{\frac{\Lambda_{5}\;Pr\;}{\Lambda_{6}}\;\frac{\;s^{1-\beta }}{{\left(1-\alpha s^{-\alpha }\right)}^{\gamma }}}-1}\frac{e^{-y\sqrt{\frac{\Lambda_{5}\;Pr\;}{\Lambda_{6}}\;\frac{\;s^{1-\beta }}{{\left(1-\alpha s^{-\alpha }\right)}^{\gamma }}}}}{s}$$

Table 3 and Table 4 show that the Laplace inverse of the aforementioned thermal profile solution will be numerically investigated using the Stehfest and Tzou techniques. 

### 4.2. Solution of the Concentration Profile

For the solution of the concentration of the boundary layers, using the LT on the non-dimensionalized and converted with the leading Equations (8) and (13), and using the result of Equation (11), the solution of ${\bar{C}}_{\left(y,s\right)}$ overcomes that.

(19) $$Sc\;s\;{\bar{C}}_{\left(y,s\right)}=-\frac{\partial {\bar{J}}_{\left(y,s\right)}}{\partial y}$$

(20) $${\bar{J}}_{\left(y,s\right)}=-s^{\beta }{\left(1-\alpha s^{-\alpha }\right)}^{\gamma }\frac{\partial {\bar{J}}_{\left(y,s\right)}}{\partial y}$$

(21) $${\bar{C}}_{\left(0,s\right)}=\frac{1}{s};\;\;\;{\bar{C}}_{\left(y,s\right)}\to 0,\;y\to \infty$$

Inserting Equation (20) into (19) and applying the above-transformed conditions, the concentration field will be

(22) $${\bar{C}}_{\left(\xi ,s\right)}=\frac{1}{s}e^{-\xi \sqrt{\frac{Sc\;s^{1-\beta }}{{\left(1-\alpha s^{-\alpha }\right)}^{\gamma }}}}$$

Again the Laplace inverse of the above-attained solution will be performed, numerically, in Table 3 and Table 4.

### 4.3. Solution for the Velocity Profile

This section examines the semi-analytical solution of the momentum profile with its physically transformed conditions. Bearing in mind the result of Equation (13), employing the LT on Equation (6), the non-homogeneous ordinary differential equation from the momentum field will be as follows

(23) $$\Lambda_{1}\frac{\partial^{2}{\bar{w}}_{\left(y,s\right)}}{\partial y^{2}}-\left(\Lambda_{2}M\;sin\left(\theta_{1}\right)+\frac{\Lambda_{1}}{K}+\Lambda_{o}\left(s+\beta_{1}\right)\right){\bar{w}}_{\left(y,s\right)}=-\Lambda_{3}Cos\left(\theta_{2}\right){\bar{T}}_{\left(y,s\right)}-\Lambda_{4}Cos\left(\theta_{2}\right){\bar{C}}_{\left(y,s\right)}$$

$${\bar{w}}_{\left(0,s\right)}-{\frac{\partial {\bar{w}}_{\left(y,s\right)}}{\partial y}|}_{y=0}=F\left(s\right);\;\;\;{\bar{w}}_{\left(y,s\right)}\to 0\;as\;y\to \infty$$

By interpolating the above-considered conditions and simplifying Equation (20), the momentum profile will be

(24) $${\bar{w}}_{\left(y,s\right)}=\frac{1}{1+h\sqrt{\frac{1}{\Lambda_{1}}\left(\Pi_{3}+\Lambda_{o}s\right)}}\left(\frac{\Pi_{5}}{\Lambda_{1}s\left(\sqrt{\frac{\Pi_{4}\;s}{s^{\beta }{\left(1-\alpha s^{-\alpha }\right)}^{\gamma }}}-1\right)}\;\frac{1+h\sqrt{\frac{\Lambda_{5}Pr}{\Lambda_{6}}\frac{s}{s^{\beta }{\left(1-\alpha s^{-\alpha }\right)}^{\gamma }}}}{\left(\frac{\Pi_{4}\;s}{s^{\beta }{\left(1-\alpha s^{-\alpha }\right)}^{\gamma }}\right)-\frac{1}{\Lambda_{1}}\left(\Pi_{3}+\Lambda_{o}s\right)}\;+\frac{\Pi_{6}}{\Lambda_{1}s}\;\frac{1+h\sqrt{\frac{Sc\;s}{s^{\beta }{\left(1-\alpha s^{-\alpha }\right)}^{\gamma }}}}{\left(\frac{Sc\;s}{s^{\beta }{\left(1-\alpha s^{-\alpha }\right)}^{\gamma }}\right)-\frac{1}{\Lambda_{1}}\left(\Pi_{3}+\Lambda_{o}s\right)}+F\left(s\right)\right)e^{-y\sqrt{\frac{1}{\Lambda_{1}}\left(\Pi_{3}+\Lambda_{o}s\right)}}\;-\frac{\Pi_{5}}{\Lambda_{1}s\left(\sqrt{\frac{\Pi_{4}\;s}{s^{\beta }{\left(1-\alpha s^{-\alpha }\right)}^{\gamma }}}-1\right)}\;\frac{e^{-y\sqrt{\frac{\Pi_{4}\;s}{s^{\beta }{\left(1-\alpha s^{-\alpha }\right)}^{\gamma }}}}}{\left(\frac{\Pi_{4}\;s}{s^{\beta }{\left(1-\alpha s^{-\alpha }\right)}^{\gamma }}\right)-\frac{1}{\Lambda_{1}}\left(\Pi_{3}+\Lambda_{o}s\right)}\;-\frac{\Pi_{6}}{\Lambda_{1}s}\;\frac{e^{-y\sqrt{\frac{Sc\;s}{s^{\beta }{\left(1-\alpha s^{-\alpha }\right)}^{\gamma }}}}}{\left(\frac{Sc\;s}{s^{\beta }{\left(1-\alpha s^{-\alpha }\right)}^{\gamma }}\right)-\frac{1}{\Lambda_{1}}\left(\Pi_{3}+\Lambda_{o}s\right)}$$

where

$$\Pi_{1}=\Lambda_{2}M\;Sin\left(\theta_{1}\right),\;\;{\;\Pi }_{2}=\frac{\Lambda_{1}}{K}+\Lambda_{o}\beta_{1},\;\;{\;\Pi }_{3}=\Pi_{1}+\Pi_{2},$$

$${\;\Pi }_{4}=\frac{\Lambda_{5}Pr}{\Lambda_{6}},\;\;{\;\Pi }_{5}=\Lambda_{3}Gr\;Cos\left(\theta_{2}\right),\;\;{\;\Pi }_{6}=\Lambda_{4}Gm\;Cos\left(\theta_{2}\right)$$

We can’t find the inverse transform analytically in the complex transform spaces for several real-world applications. To produce the inverse Laplace, authors have also employed specific numerical techniques. Many scholars have successfully applied Stehfest’s technique and Tzou’s [58] for the numerical Laplace method, to solve the fractional differential equations, efficiently and effectively. The formula for the Stehfest and Tzou algorithms is as follows, respectively:

$$f\left(y,t\right)=\frac{ln\left(2\right)}{t}\overset{2N}{\underset{n=1}{\sum }}h_{n}\bar{f}\left(y,n\frac{ln\left(2\right)}{t}\right)$$

where N is a positive integer.

$$h_{n}={\left(-1\right)}^{n+\frac{N}{2}}\overset{min\left(p,N\right)}{\underset{r=\left[\frac{p+1}{2}\right]}{\sum }}\frac{r^{N}\left(2r\right)!}{\left(N-r\right)!r!\;\left(r-1\right)!\;\left(p-r\right)!\;\left(2r-p\right)!}$$

and

$$f\left(y,t\right)=\frac{e^{4.7}}{t}\left[\frac{1}{2}\bar{f}\left(r,\frac{4.7}{t}\right)+Re\left\{\overset{N}{\underset{j=1}{\sum }}{\left(-1\right)}^{j}\bar{f}\left(r,\frac{4.7+k\pi i}{t}\right)\right\}\right]$$

**Limiting Case:**

If, in the attained solution of the momentum field Equation (21), the physical quantities$\;Gm=Sc=K=0,\;\theta_{1}=\frac{\pi }{2}\;\;$become zero, then

(25) $${\bar{w}}_{\left(y,s\right)}=\frac{1}{1+h\sqrt{\frac{1}{\Lambda_{1}}\left(\Pi_{3}+\Lambda_{o}s\right)}}\left(\frac{\Pi_{5}}{\Lambda_{1}s\left(\sqrt{\frac{\Pi_{4}\;s}{s^{\beta }{\left(1-\alpha s^{-\alpha }\right)}^{\gamma }}}-1\right)}\;\frac{1+h\sqrt{\frac{\Pi_{4}\;s}{s^{\beta }{\left(1-\alpha s^{-\alpha }\right)}^{\gamma }}}}{\left(\frac{\Pi_{4}\;s}{s^{\beta }{\left(1-\alpha s^{-\alpha }\right)}^{\gamma }}\right)-\frac{1}{\Lambda_{1}}\left(\Pi_{3}+\Lambda_{o}s\right)}\;+F\left(s\right)\right)e^{-y\sqrt{\frac{1}{\Lambda_{1}}\left(\Pi_{3}+\Lambda_{o}s\right)}}-\frac{\Pi_{5}}{\Lambda_{1}s\left(\sqrt{\frac{\Pi_{4}\;s}{s^{\beta }{\left(1-\alpha s^{-\alpha }\right)}^{\gamma }}}-1\right)}\;\frac{e^{-y\sqrt{\frac{\Pi_{4}\;s}{s^{\beta }{\left(1-\alpha s^{-\alpha }\right)}^{\gamma }}}}}{\left(\frac{\Pi_{4}\;s}{s^{\beta }{\left(1-\alpha s^{-\alpha }\right)}^{\gamma }}\right)-\frac{1}{\Lambda_{1}}\left(\Pi_{3}+\Lambda_{o}s\right)}$$

The above-attained solution of the velocity profile is quite similar to the attained solution by Saqib et al. [45]. Furthermore, the Prabhakar fractional constraints β, γ can be taken as zero for the generalized results of the governed equations. 

**Special Cases:**

The following are essential exceptional cases, with a technical significance that is widely recognized and well defined in the existing literature to understand the problem better.

**Case 1:** ***f*(*t*) = *Sin*(*ωt*)**

In the first particular case, consider the function f(t)=Sin(ωt), in which ω signifies the amplitude of the oscillating plate, then the momentum profile for this case will be

(26) $${\bar{w}}_{\left(y,s\right)}=\frac{1}{1+h\sqrt{\frac{1}{\Lambda_{1}}\left(\Pi_{3}+\Lambda_{o}s\right)}}\left(\frac{\Pi_{5}}{\Lambda_{1}s\left(\sqrt{\frac{\Pi_{4}\;s}{s^{\beta }{\left(1-\alpha s^{-\alpha }\right)}^{\gamma }}}-1\right)}\;\frac{1+h\sqrt{\frac{\Lambda_{5}Pr}{\Lambda_{6}}\frac{s}{s^{\beta }{\left(1-\alpha s^{-\alpha }\right)}^{\gamma }}}}{\left(\frac{\Pi_{4}\;s}{s^{\beta }{\left(1-\alpha s^{-\alpha }\right)}^{\gamma }}\right)-\frac{1}{\Lambda_{1}}\left(\Pi_{3}+\Lambda_{o}s\right)}\;+\frac{\Pi_{6}}{\Lambda_{1}s}\;\frac{1+h\sqrt{\frac{Sc\;s}{s^{\beta }{\left(1-\alpha s^{-\alpha }\right)}^{\gamma }}}}{\left(\frac{Sc\;s}{s^{\beta }{\left(1-\alpha s^{-\alpha }\right)}^{\gamma }}\right)-\frac{1}{\Lambda_{1}}\left(\Pi_{3}+\Lambda_{o}s\right)}+\frac{\omega }{\omega^{2}+s^{2}}\right)e^{-y\sqrt{\frac{1}{\Lambda_{1}}\left(\Pi_{3}+\Lambda_{o}s\right)}}\;-\frac{\Pi_{5}}{\Lambda_{1}s\left(\sqrt{\frac{\Pi_{4}\;s}{s^{\beta }{\left(1-\alpha s^{-\alpha }\right)}^{\gamma }}}-1\right)}\;\frac{e^{-y\sqrt{\frac{\Pi_{4}\;s}{s^{\beta }{\left(1-\alpha s^{-\alpha }\right)}^{\gamma }}}}}{\left(\frac{\Pi_{4}\;s}{s^{\beta }{\left(1-\alpha s^{-\alpha }\right)}^{\gamma }}\right)-\frac{1}{\Lambda_{1}}\left(\Pi_{3}+\Lambda_{o}s\right)}\;-\frac{\Pi_{6}}{\Lambda_{1}s}\;\frac{e^{-y\sqrt{\frac{Sc\;s}{s^{\beta }{\left(1-\alpha s^{-\alpha }\right)}^{\gamma }}}}}{\left(\frac{Sc\;s}{s^{\beta }{\left(1-\alpha s^{-\alpha }\right)}^{\gamma }}\right)-\frac{1}{\Lambda_{1}}\left(\Pi_{3}+\Lambda_{o}s\right)}$$

**Case 2:** ***f*(*t*) = *Cos*(*ωt*)**

In the second particular case, consider the function f(t)=Cos(ωt), then the momentum profile for this case will be

(27) $${\bar{w}}_{\left(y,s\right)}=\frac{1}{1+h\sqrt{\frac{1}{\Lambda_{1}}\left(\Pi_{3}+\Lambda_{o}s\right)}}\left(\frac{\Pi_{5}}{\Lambda_{1}s\left(\sqrt{\frac{\Pi_{4}\;s}{s^{\beta }{\left(1-\alpha s^{-\alpha }\right)}^{\gamma }}}-1\right)}\;\frac{1+h\sqrt{\frac{\Lambda_{5}Pr}{\Lambda_{6}}\frac{s}{s^{\beta }{\left(1-\alpha s^{-\alpha }\right)}^{\gamma }}}}{\left(\frac{\Pi_{4}\;s}{s^{\beta }{\left(1-\alpha s^{-\alpha }\right)}^{\gamma }}\right)-\frac{1}{\Lambda_{1}}\left(\Pi_{3}+\Lambda_{o}s\right)}\;+\frac{\Pi_{6}}{\Lambda_{1}s}\;\frac{1+h\sqrt{\frac{Sc\;s}{s^{\beta }{\left(1-\alpha s^{-\alpha }\right)}^{\gamma }}}}{\left(\frac{Sc\;s}{s^{\beta }{\left(1-\alpha s^{-\alpha }\right)}^{\gamma }}\right)-\frac{1}{\Lambda_{1}}\left(\Pi_{3}+\Lambda_{o}s\right)}+\frac{q}{\omega^{2}+s^{2}}\right)e^{-y\sqrt{\frac{1}{\Lambda_{1}}\left(\Pi_{3}+\Lambda_{o}s\right)}}\;-\frac{\Pi_{5}}{\Lambda_{1}s\left(\sqrt{\frac{\Pi_{4}\;s}{s^{\beta }{\left(1-\alpha s^{-\alpha }\right)}^{\gamma }}}-1\right)}\;\frac{e^{-y\sqrt{\frac{\Pi_{4}\;s}{s^{\beta }{\left(1-\alpha s^{-\alpha }\right)}^{\gamma }}}}}{\left(\frac{\Pi_{4}\;s}{s^{\beta }{\left(1-\alpha s^{-\alpha }\right)}^{\gamma }}\right)-\frac{1}{\Lambda_{1}}\left(\Pi_{3}+\Lambda_{o}s\right)}\;-\frac{\Pi_{6}}{\Lambda_{1}s}\;\frac{e^{-y\sqrt{\frac{Sc\;s}{s^{\beta }{\left(1-\alpha s^{-\alpha }\right)}^{\gamma }}}}}{\left(\frac{Sc\;s}{s^{\beta }{\left(1-\alpha s^{-\alpha }\right)}^{\gamma }}\right)-\frac{1}{\Lambda_{1}}\left(\Pi_{3}+\Lambda_{o}s\right)}$$

**Case 3:** ***f*(*t*) = *te^t^***

In the third particular case, consider the function $f\left(t\right)=te^{t}\;$having its Laplace$\;F\left(s\right)=\frac{1}{{\left(s-1\right)}^{2}}\;,\;$then the momentum field for this case will be

(28) $${\bar{w}}_{\left(y,s\right)}=\frac{1}{1+h\sqrt{\frac{1}{\Lambda_{1}}\left(\Pi_{3}+\Lambda_{o}s\right)}}\left(\frac{\Pi_{5}}{\Lambda_{1}s\left(\sqrt{\frac{\Pi_{4}\;s}{s^{\beta }{\left(1-\alpha s^{-\alpha }\right)}^{\gamma }}}-1\right)}\;\frac{1+h\sqrt{\frac{\Lambda_{5}Pr}{\Lambda_{6}}\frac{s}{s^{\beta }{\left(1-\alpha s^{-\alpha }\right)}^{\gamma }}}}{\left(\frac{\Pi_{4}\;s}{s^{\beta }{\left(1-\alpha s^{-\alpha }\right)}^{\gamma }}\right)-\frac{1}{\Lambda_{1}}\left(\Pi_{3}+\Lambda_{o}s\right)}\;+\frac{\Pi_{6}}{\Lambda_{1}s}\;\frac{1+h\sqrt{\frac{Sc\;s}{s^{\beta }{\left(1-\alpha s^{-\alpha }\right)}^{\gamma }}}}{\left(\frac{Sc\;s}{s^{\beta }{\left(1-\alpha s^{-\alpha }\right)}^{\gamma }}\right)-\frac{1}{\Lambda_{1}}\left(\Pi_{3}+\Lambda_{o}s\right)}+\frac{1}{{\left(s-1\right)}^{2}}\right)e^{-y\sqrt{\frac{1}{\Lambda_{1}}\left(\Pi_{3}+\Lambda_{o}s\right)}}\;-\frac{\Pi_{5}}{\Lambda_{1}s\left(\sqrt{\frac{\Pi_{4}\;s}{s^{\beta }{\left(1-\alpha s^{-\alpha }\right)}^{\gamma }}}-1\right)}\;\frac{e^{-y\sqrt{\frac{\Pi_{4}\;s}{s^{\beta }{\left(1-\alpha s^{-\alpha }\right)}^{\gamma }}}}}{\left(\frac{\Pi_{4}\;s}{s^{\beta }{\left(1-\alpha s^{-\alpha }\right)}^{\gamma }}\right)-\frac{1}{\Lambda_{1}}\left(\Pi_{3}+\Lambda_{o}s\right)}\;-\frac{\Pi_{6}}{\Lambda_{1}s}\;\frac{e^{-y\sqrt{\frac{Sc\;s}{s^{\beta }{\left(1-\alpha s^{-\alpha }\right)}^{\gamma }}}}}{\left(\frac{Sc\;s}{s^{\beta }{\left(1-\alpha s^{-\alpha }\right)}^{\gamma }}\right)-\frac{1}{\Lambda_{1}}\left(\Pi_{3}+\Lambda_{o}s\right)}$$

**Validity of the Fractional Model**

**Figure 2:**
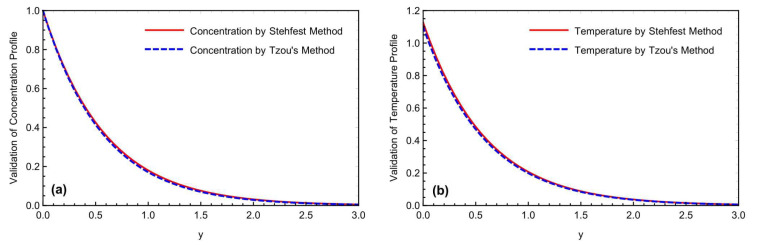
(**a,b**) Comparison of the numerical schemes for the concentration and temperature fields.

**Figure 3:**
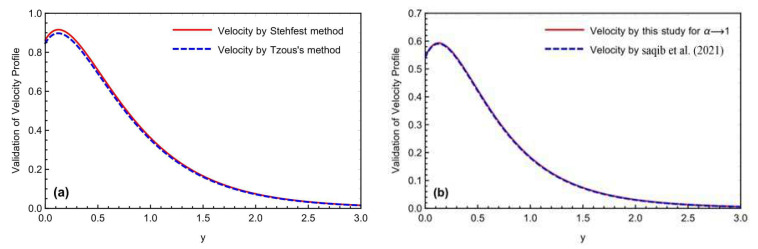
(**a,b**) Comparison of the numerical schemes and Saqib et al. [42], for the momentum field.

Both numerical approaches, Stehfest’s and Tzou’s, were compared by drawing Figure 2a,b for the concentration and temperature profiles. There is a slight overlap of the findings between the two curves. Figure 3a,b compares both numerical methods and solutions for the velocity field, using the Prabhakar fractional methodology with Saqib et al. [42]. The simulations obtained by employing the Prabhakar fractional model have a good accuracy, compared to Saqib et al.’s study [42].

## 5. Results with Discussion

**Figure 4:**
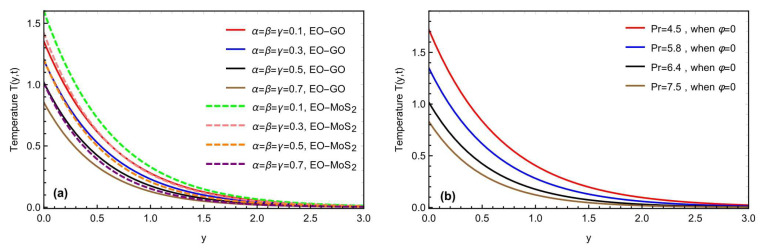
(**a,b**) Consequence of the fractional constraints and Pr on the temperature field.

**Figure 5:**
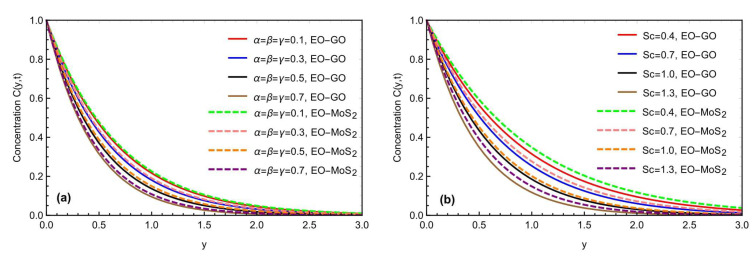
(**a**,**b**) Consequence of the fractional constraints and Sc on the concentration field.

**Figure 6:**
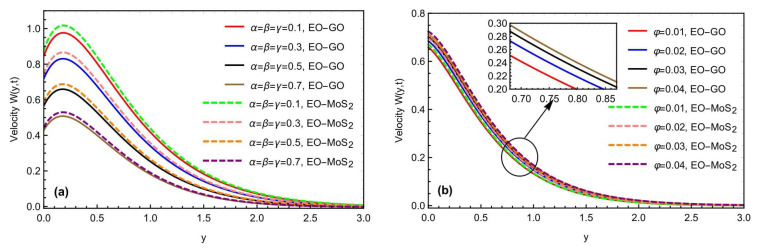
(**a**,**b**) Consequence of α,β,γ, and φ on the momentum profile with$\;Sc=1.2,Gr=10.4,Gm=8.2,M=1.5,K=0.7,h=0.5,\;\;\theta_{1}=\theta_{2}=\frac{\pi }{4},t=0.9$.

**Figure 7:**
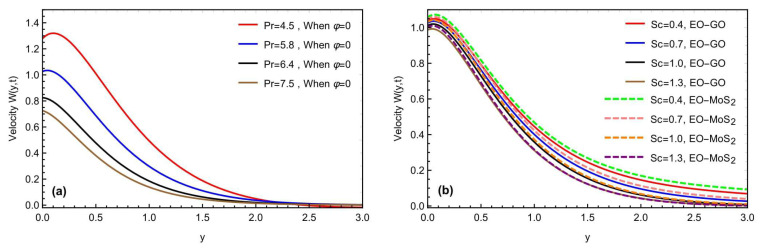
(**a**,**b**) Consequence of Pr and Sc on the momentum profile with$\;\alpha =\beta =\gamma =0.8,Gr=10.4,Gm=8.2,M=1.5,K=0.7,h=0.5,\;\;\theta_{1}=\theta_{2}=\frac{\pi }{4},t=0.9$.

**Figure 8:**
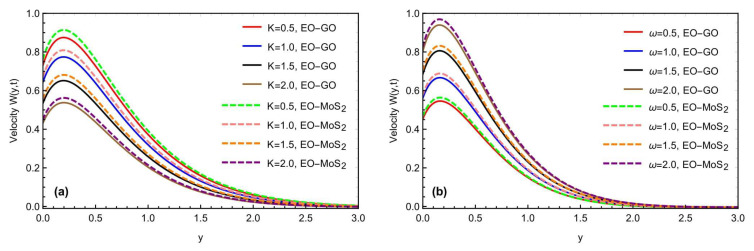
(**a**,**b**) Consequence of K and ω on the momentum profile with$\;\alpha =\beta =\gamma =0.8,Sc=1.2,M=1.5,\;\mathrm{Gr}=10.4,\;\mathrm{Gm}=8.2,K=0.7,h=0.5,\theta_{1}=\theta_{2}=\frac{\pi }{4},t=0.9$.

**Figure 9:**
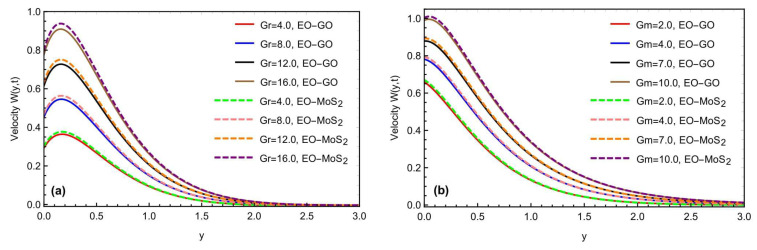
(**a,b**) Consequence of Gr and Gm on the momentum profile with$\;\alpha =\beta =\gamma =0.8,Sc=1.2,M=1.5,K=0.7,h=0.5,\theta_{1}=\theta_{2}=\frac{\pi }{4},t=0.9$.

**Figure 10:**
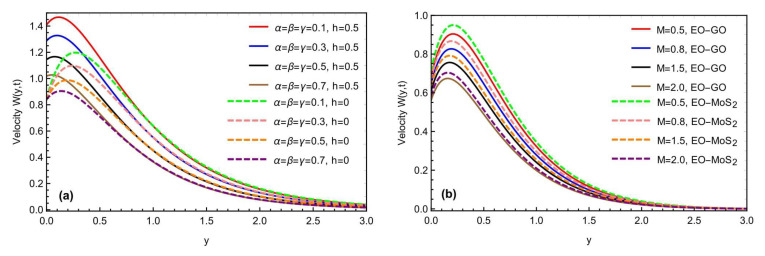
(**a**,**b**) Consequence of h and M on the momentum profile with$\;\alpha =\beta =\gamma =0.8,Sc=1.2,Gr=10.4,Gm=8.2,K=0.7,\;\;\theta_{1}=\theta_{2}=\frac{\pi }{4},t=0.9$.

**Figure 11:**
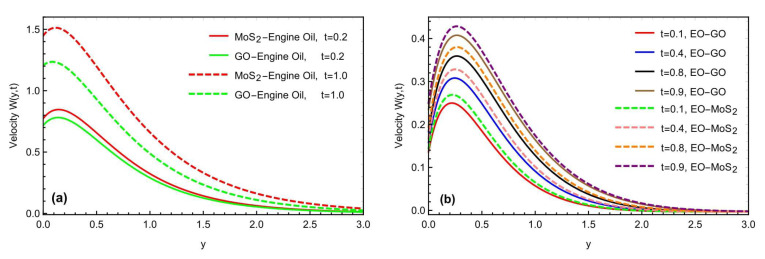
(**a**,**b**) Consequence of time t and comparison of the different nanofluids with the different nanoparticles.

The mixed convection Brinkman-type nanofluid model’s thermal study, due to an oscillating inclined plate in an applied magnetic field, is examined using fractional simulations. Different nanoparticles, i.e., molybdenum disulfide $\left(MOS_{2}\right)\;$and graphene-oxide (GO) are utilized for the nanofluid with a base engine oil fluid. A close thermal inspection of the modified nanofluid model is provided using molybdenum disulfide and graphene oxide nanoparticles with a viscoelastic base fluid. In the preceding part, the Prabhakar fractional derivative framework was successfully executed for the solution of the governed fractional equations. Some exceptional cases for the momentum profile are also described, improving the flowing fluid’s physical significance. The physical impact of the different constraints on the thermal, concentration, and momentum profiles are also analyzed graphically in Figure 4, Figure 5, Figure 6, Figure 7, Figure 8, Figure 9, Figure 10 and Figure 11, with different nanoparticles and different ranges, such as 0.1<α, β, γ<0.9, 4<Gr<16, 0.01<φ<0.04, 0.5<ω<2.0, 2.0<Gm<10, 0.5<K<2.0, 0.5<M<2.0 and 0.1<Sc<1.5. This section outlined the thermal dynamics of the flow model when the flow parameters were varied.

Figure 4a,b shows how the fractional restrictions (α, β, γ) and the Prandtl number Pr affect the temperature profile. The thermal properties of the molybdenum disulfide-engine oil $\left(MOS_{2}-EO\right)$ and the graphene oxide-engine oil (GO−EO) nanofluids are compared. By changing (α, β, γ), the temperature profile for both $MOS_{2}-EO$ and (GO−EO) nanomaterial suspensions showed a declining tendency. The thermal profile nearer the plate is maximal for the lower values of the fractional parameters, while the most significant fall in the profile is seen for the bigger values. The higher values of (α, β, γ) related to the thickness of the thermal and momentum boundary layers represent the physical point. The higher values of reducing (α, β, γ) the thickness of the thermal boundary layer, result in a declining trend in the temperature and velocity profiles. Moreover, the improvement in thermal rate, due to $MOS_{2}-EO$ is more progressive, as compared to GO−EO, due to the physical characteristics of the selected nanoparticles. Figure 4b conveys the effects of Pr, showing a declining change in the temperature field. Mathematically, as Pr is the ratio of the kinematic viscosity to the thermal diffusivity and, physically, varying the value of the Prandtl number, the thermal conductivity reduces, and the fluid becomes thicker, due to which the temperature reduces, as depicted in Figure 4b. Figure 5a,b indicates the impact of the fractional constraints α,β,γ, and the Schmidt number *Sc* on the boundary layer concentration for both types of nanofluids$\;\left(MOS_{2}-EO\right)\;$and (GO−EO). Again, similar to the thermal profile, the concentration field also decays by increasing the fractional constraints and Schmidt number values. Physically, the molecular diffusivity will decrease with the Schmidt number values’ enhancement. Furthermore, with the thermal field, the concentration profile also declares more values in the graphical representation for the molybdenum disulfide$\;\left(MOS_{2}\right)\;$based nanofluid, as compared to the graphene oxide (GO) mixed suspension. 

Figure 6a,b highlights the physical impact of the fractional constraints α,β,γ and the volume fraction on the momentum field for both the molybdenum disulfide-engine oil $\left(MOS_{2}-EO\right)$ and graphene oxide-engine oil (GO−EO) type nanofluids. Increasing the values of (α,β,γ), lowers the mobility of the EO-based nanofluid, due to a decrease in the thickness of the momentum barrier layer. Assigning the variation in the fractional parameters, the fluid motion slows down. Moreover, with the variation in the volume fraction parameters, the velocity profile is improved, due to the effective density of the considered nanoparticles. The fluid velocity is also improved for the$\;\left(MOS_{2}\right)\;$based nanoparticles, as compared to the (*GO*) suspension, due to the heat conduction. As the volume fraction increases, the fluid becomes more viscous, indicating that the GO nanoparticles’ fluid velocity will decrease, owing to the heat conduction. These novel observations may present many applications in improving the thermal capacitance of various engineering and industrial processes. Figure 7a,b predicts the influence of the Prandtl number Pr and the Schmidt number Sc on the momentum profile of the Brinkman-type nanofluid. The flowing rate is observed as the decreasing value for both parameters Pr and Sc. As the enhancement in Pr creates some more hinderances and increases the flowing fluid viscosity, it results in a decrement in the fluid velocity, and the fluid flows more slowly. Similarly, similar to the impact of the Prandtl number, the Schmidt number also slows down the fluid motion, due to a decrement in the diffusivity rate of the flowing fluid. It is concluded that the velocity profile of the nanoparticles (GO) decreases while approaching the plate, but the velocity of the nanoparticles (MoS_2_) rises owing to the nanoparticle features. 

The graphical impact of the porosity parameter K and the effects of the oscillations of the inclined plate, is represented in Figure 8a,b. For the general function f(t), we have considered the function Sin(ωt) as a particular function, where ω represents the frequency of the oscillations. Moreover, this can be seen that the fluid flow moves fast with the increment in the frequency of the oscillations. Figure 9a,b depicts the graphical behavior of the heat Grashof number Gr and the mass Grashof number Gm on a velocity profile. The velocity profile has been enhanced with large values of Gr and Gm. Physically, the heat Grashof number Gr creates natural convection, owing to the buoyant force, and an increase in the Gm improves the buoyancy forces, increasing the fluid velocity. This is the weighted average of the buoyancy and viscous forces. A higher Gr generates an increase in the buoyant forces, which causes the generated flows to increase. Figure 10a,b depicts the impact of the slip parameter h and the magnetic number M on the fluid velocity. The velocity field was reduced by raising the magnetic parameter value. Physically, an increase in the magnetic field results in an increase in the Lorentz force, which decreases the velocity of the fluid. Furthermore, the maximum effect of the Lorentz forces is when the angle of inclination of the applied magnetics is at the right angle to the oscillating plate. M is a dimensionless number associated with the Lorentz force, which opposes the nanofluid velocity. The more excellent the M, the greater the Lorentz force, which resists motion. As a result, the velocity was slowed in both cases of the engine oil-based nanofluid with the increasing M. Similarly, the inclination of a magnetic field reduces the influence of M on the Lorentz force. The Lorentz force has the most significant impact at$\;\theta_{1}=\pi /2$ (normal magnetic field), as seen in the figures. While the slip parameter h improved, the fluid motion and momentum field increased with the enhancement in the slip factor, as predicted in the figures. Figure 11a,b predicts the comparative change in the flow rate with the interaction of all types of nanoparticles $MOS_{2}\;and\;Go$ with the engine oil as based liquid and the effects of time t on the momentum field. It is interesting to note that the interaction of the $MOS_{2}$ nanoparticles are more practical in improving the flowing fluid rate, as compared to the  GO nanoparticles. The addition of the different nanoparticles means an enhancement in the fluid density, increasing the flowing nanofluid’s thickness. Therefore, the  (GO) nanoparticle-based suspension of the nanofluid has a lower flowing rate, as compared to the$\;\left(MoS_{2}\right)\;$nanoparticles mixed with the nanofluid. Furthermore, as illustrated in Figure 11b, the thickness of the boundary layer grows thicker than the thickness of the momentum boundary layer with time, thus enhancing the fluid motion.

Finally, the numerical analysis of the temperature, concentration, and velocity profiles by Stehfest’s and Tzou’s numerical schemes, is analyzed in Table 3. To validate our numerical inverse Laplace, transform solutions using Stehfest’s and Tzou’s methods, we performed numerical calculations for the temperature and velocity fields, which were found to be in good agreement, as shown in Table 3. The temperature profile, concentration, and momentum fields declined as they moved away from the inclined plate with the increasing y and became zero for y→∞, which signifies the boundary conditions. Furthermore, in the numerical analysis of both Stehfest’s and Tzou’s algorithms, very closed values of all governed parameters by the different numerical techniques, also validate this study. At instant rises, the decreasing change in the heat transmission and mass transfer is progressive. According to Table 4, the local Nusselt number and the wall shear force decreased as it increased, indicating a significant agreement for the temperature profile. Table 5 also shows the percentage difference in the momentum field analysis.

## 6. Conclusions

The fractional-supported Brinkman-type nanofluid was examined using different nanoparticles and an oscillating inclined poured plate. Molybdenum disulfide and graphene oxide nanoparticles with a viscoelastic base liquid are investigated to increase the thermal performance. The motivations for implementing the Prabhakar-like fractional model are justified, as this fractional algorithm contains modern definitions without any restriction of singularities. The correctness of the fractional scheme is checked by setting up a comparison task with another fractional approach, and several unique and limiting circumstances are given to obtain the physical insight into the examined fractional model. Finally, the thermal determination of the used molybdenum-disulfide$\;\left(MoS_{2}\right)\;$and graphene-oxide (GO) nanoparticles are depicted in comparison. The following are the most critical findings from the present model:

- The fractional parameters α,β,γ declined the thermal, concentration, and momentum profiles for the$\;MoS_{2}-EO\;$and GO−EO nanoparticle suspension and the rate decrement are thicker as the time instants increase.
- The heat transfer rate for both nanoparticles susceptible to the engine oil base material may be regulated for the specific Prandtl number values.
- Because of the thermal conductivity factor, the thermal performances of the molybdenum disulfide $MoS_{2}$ nanoparticles with engine oil base fluid are more progressive than the graphene oxide GO nanoparticles.
- The changing velocity is caused by the variations in the heat and mass Grashof constants for the molybdenum disulfide engine oil and the graphene oxide engine oil suspensions.
- Using the Prabhakar operators with the fractional coefficient’s parameter settings, might help to select an appropriate computational formula that produces a good consistency between the experimental and theoretical values.
- In the graphical comparison of the attained solution of the momentum profile with Saqib et al. [42], the overlapping of both curves validates the attained results of this study.
- Implementing the Prabhakar fractional derivative technique is also a powerful tool to simulate the analytical expressions for any coupled and nonlinear partial differential system.
- The patterns and characteristics of all physical flow metrics coincide perfectly with the published studies.
- The overlaying of both curves in assessing both numerical techniques confirms the obtained solutions of the governed equations.

Our computational effort has effectively clarified the parametric implications of the flow of two different nanoparticle phases. The results for the two different nanoparticles show significant monotonic differences. This study might be expanded to three or more hybrid phases to identify which hybrid phase is the most effective.

## Figures and Tables

**Table 1:** Model for the thermophysical properties of the nanofluid quantities [54].

Thermal Features	Nanofluid
Density	$\frac{\rho_{nf}}{\rho_{f}}=\left(1-\varphi \right)+\varphi \frac{\rho_{s}}{\rho_{f}}\rho_{f}$
Dynamic Viscosity	$\frac{\mu_{nf}}{\mu_{f}}=\frac{1}{{\left(1-\varphi \right)}^{2.5}}$
Thermal Expansion Coefficient	$\frac{{\left(\rho \beta_{T}\right)}_{nf}}{{\left(\rho \beta_{T}\right)}_{f}}=\left(1-\varphi \right)+\varphi \frac{{\left(\rho \beta_{T}\right)}_{s}}{{\left(\rho \beta_{T}\right)}_{f}}$
Electrical Conductivity	$\frac{\sigma_{nf}}{\sigma_{f}}=\left(1+\frac{3\left(\frac{\sigma_{s}}{\sigma_{f}}-1\right)\varphi }{\left(\frac{\sigma_{s}}{\sigma_{f}}+2\right)-\left(\frac{\sigma_{s}}{\sigma_{f}}-1\right)\varphi }\right)$
Concentration Expansion Coefficient	$\frac{{\left(\rho \beta_{C}\right)}_{nf}}{{\left(\rho \beta_{C}\right)}_{f}}=\left(1-\varphi \right)+\varphi \frac{{\left(\rho \beta_{C}\right)}_{s}}{{\left(\rho \beta_{C}\right)}_{f}}$
Thermal Conductivity	$\frac{k_{nf}}{k_{f}}=\left(\frac{k_{s}+\left(n-1\right)k_{f}-\left(n-1\right)\left(k_{f}-k_{s}\right)\varphi }{k_{s}+\left(n-1\right)k_{f}+\left(k_{f}-k_{s}\right)\varphi }\right)$
Heat Capacitance	$\frac{{\left(\rho C_{p}\right)}_{nf}}{{\left(\rho C_{p}\right)}_{f}}=\left(1-\varphi \right)+\varphi \frac{{\left(\rho C_{p}\right)}_{s}}{{\left(\rho C_{p}\right)}_{f}}$

**Table 2:** Thermal properties of the base liquids and nanoparticles [55].

Material	Engine Oil	MoS_2_	GO
$\rho \;\left(\mathrm{kg}/m^{3}\right)$	884	5060	1800
$C_{p}\;\left(J/\mathrm{kg}\;K\right)$	1910	397.21	717
*k* (W/m K)	0.144	904.4	5000
$\beta_{T}\;\left(K^{-1}\right)$	$70\times 10^{-5}$	$2.8424\times 10^{-5}$	$0.284\times 10^{-5}$
$\beta_{C\;}\left(m^{2}h^{-1}\right)$	$165.5\times 10^{-5}$	$2.05\times 10^{-5}$	$0.657\times 10^{-5}$
σ (Ωm)	$2.09\times 10^{-4}$	$2.09\times 10^{-4}$	$1\times 10^{7}$
Pr	233	-	-

**Table 3:** Numerical variation of the thermal, concentration, and momentum fields.

*y*	Temp by Stehfest	Temp by Stehfest	Conc by Tzou	Conc by Tzou	Vel by Stehfest	Vel by Tzou
0.1	0.702	0.733	0.795	0.801	0.684	0.681
0.3	0.480	0.489	0.501	0.514	0.553	0.547
0.5	0.328	0.326	0.314	0.328	0.414	0.407
0.7	0.224	0.217	0.196	0.209	0.296	0.289
0.9	0.153	0.144	0.121	0.132	0.206	0.199
1.1	0.104	0.095	0.074	0.083	0.141	0.134
1.3	0.071	0.063	0.044	0.052	0.095	0.089
1.5	0.049	0.041	0.026	0.032	0.063	0.059
1.7	0.033	0.027	0.015	0.022	0.042	0.038
1.9	0.023	0.018	0.009	0.012	0.028	0.024

**Table 4:** Numerical influence of different constraints on the Nusselt number, Sherwood number, and the skin friction.

α	φ	t	Pr/Sc	Nu	Sh	$C_{f}$
0.1	0.02	0.6	4.7	1.856	1.640	1.230
0.2	0.02	0.6	4.7	1.773	1.709	1.105
0.3	0.02	0.6	4.7	1.708	1.776	1.012
0.2	0.01	0.6	4.7	1.521	1.694	0.380
0.2	0.02	0.6	4.7	1.353	1.598	0.163
0.2	0.03	0.6	4.7	1.270	1.576	0.059
0.2	0.02	0.5	4.7	1.523	2.014	0.562
0.2	0.02	0.6	4.7	1.473	2.071	0.163
0.2	0.02	0.7	4.7	1.418	2.135	0.059
0.2	0.02	0.6	4.5	1.521	1.694	0.380
0.2	0.02	0.6	4.6	1.353	1.598	0.163
0.2	0.02	0.6	4.7	1.270	1.576	0.059

**Table 5:** Comparison of the momentum field with the work of Saqib et al. [42] when α,β,γ→1.

*y*	Velocity by This Study	Velocity by Saqib et al. [42]	Percentage Difference of both Velocities
0.1	0.887	0.901	0.086%
0.3	0.764	0.771	0.107%
0.5	0.604	0.608	0.127%
0.7	0.457	0.458	0.181%
0.9	0.337	0.336	0.248%
1.1	0.244	0.243	0.272%
1.3	0.176	0.174	0.267%
1.5	0.126	0.124	0.244%
1.7	0.089	0.083	0.214%
1.9	0.064	0.063	0.182%

## Data Availability

Not applicable.

## Nomenclatures

w-	Velocity [m/s]
t-	Time [s]
$U_{o}$-	Constant velocity [m/s]
T-	Temperature [K]
$T_{\infty }$-	Temperature of fluid away from the plate [K]
$T_{w}$-	Fluids temperature at the plate [K]
C-	Concentration of the fluid $\left[Kgm^{-3}\right]$
$C_{w}$-	Fluids concentration at the plate$\;\left[Kgm^{-3}\right]$
$C_{p}$-	Specific heat at constant pressure $\left[jKg^{-1}K^{-1}\right]$
g-	Acceleration due to gravity$\;\left[m/s^{2}\right]$
D-	Mass diffusion coefficient $\left[m^{2}s^{-1}\right]$
Sc-	Schmidt number []
$\beta_{1}$-	Casson fluid parameter []
h-	Slip parameter [-]
Gr-	Heat Grashof number [-]
$\upsilon_{f}$-	Kinematic viscosity [m^2^/s]
μ-	Dynamic viscosity $\left[Kgm^{-1}s^{-1}\right]$
σ-	Electrical conductivity [S/m]
$\theta_{2}$-	Angle of inclination of the plate [-]
α,β,γ Prabhakar	Fractional derivative operators [-]
$\theta_{1}$-	Angle of inclination of magnetic field [-]
Pr-	Prandtl number [-]
K-	Porosity parameter [-]
Gm-	Mass Grashof number [-]
s-	Laplace transformed variable [-]
Nu-	Nusselt number [-]
$C_{f}$-	Skin friction [-]
Note: This [-] represents the dimensionless quantity.	
